# Waste-Derived Caffeine for Green Synthesis of Rhenium Nanoparticles with Enhanced Catalytic Activity in the Hydrogenation of 4-Nitrophenol

**DOI:** 10.3390/ijms252011319

**Published:** 2024-10-21

**Authors:** Alicja Kuś, Anna Leśniewicz, Anna Dzimitrowicz, Pawel Pohl, Piotr Cyganowski

**Affiliations:** 1 Department of Analytical Chemistry and Chemical Metallurgy, Wroclaw University of Science and Technology, 27 Wybrzeze St. Wyspianskiego, 50-370 Wroclaw, Poland; 252615@student.pwr.edu.pl (A.K.); anna.lesniewicz@pwr.edu.pl (A.L.); anna.dzimitrowicz@pwr.edu.pl (A.D.); pawel.pohl@pwr.edu.pl (P.P.); 2 Department of Process Engineering and Technology of Polymer and Carbon Materials, Wroclaw University of Science and Technology, 27 Wybrzeze St. Wyspianskiego, 50-370 Wroclaw, Poland

**Keywords:** coffee grounds, caffeine extracts, nanocatalysis, nitroaromatics, reduction

## Abstract

Yearly, thousands of tons of wasted coffee grounds are produced according to high coffee consumption. Still, after the coffee brewing, wasted coffee grounds contain some amounts of caffeine (CAF). CAF, in turn, contains multiple O and N chelating atoms in its structure. These have a potential to be reductors for complexes of metals. In this context, within the present study, a set of CAF extracts derived from coffee beans and coffee grounds were obtained and then used for the one-step reduction of ReO_4_^−^ ions with no additional toxic chemicals. Within this approach, CAF was applied as a secondary, green resource for the synthesis of unique rhenium nanoparticles (ReNPs) containing Re species at 0 and +6 oxidation states. The obtained ReNPs were identified and characterized with the use of X-ray powder diffraction (XRD) and high-resolution transmission electron microscopy (HRTEM). Further, the capping and stabilization of ReNPs by CAF were verified with the aid of Fourier transformation infrared spectroscopy (FT-IR). The so-obtained “green” ReNPs were then used as a homogenous catalyst in the catalytic hydrogenation of 4-nitrophenol (4-NP). This new nanomaterial revealed a superior catalytic activity, leading to the complete reduction of 4-NP to 4-aminophenol within 40–60 min with a first-order rate constant of 0.255 min^−1^.

## 1. Introduction

**Figure 1:**
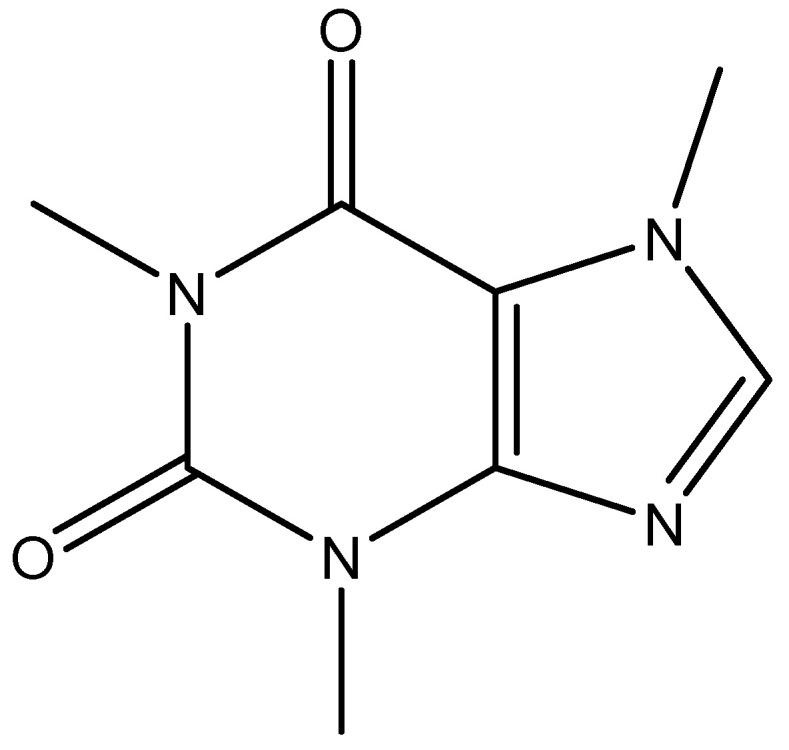
The caffeine structure.

The food industry generates thousands of tons of waste within which coffee grounds, tea leaves, or cocoa beans may be considered as an attractive, secondary source of caffeine (CAF) [1,2,3]. CAF is a compound belonging to alkaloids that may be very important for modern chemical engineering, materials science, and medicinal chemistry. CAF, containing multiple N and O chelating atoms in its chemical structure (see Figure 1 for details), offers the possibility of green approaches to the synthesis of different carriers for drug delivery systems [4] and several biologically active substances [5], as well as the production of ionic liquids for various separations [6].

The presence of chelating atoms in the CAF structure offers yet additional intriguing applications of this alkaloid. Namely, its electron donor properties may enable one to synthesize CAF–metal complexes for catalytical applications. Among very few examples, CAF was used for the formation of CAF-Au(III) [7] and CAF-Cu(I) [8] complexes, further applied for the coupling reactions and “click” chemistry of organic halides and epoxides [7,8]. These applications can be pushed even further, as the multiple free electron pairs located on O and N atoms in CAF may contribute to the synthesis of metallic nanoparticles (NPs). In this scenario, CAF may play the role of a green and sustainable capping agent for the fabrication of novel nanomaterials with catalytic properties. In this context, CAF was already used for the synthesis of biogenic CeTiO_4_ NPs [9]. The abovementioned CAF-based materials were applied in the coupling reactions of aldehydes and secondary amines [7], as well as the photocatalytic degradation of dyes [9]. All examples clearly indicate that CAF may indeed be used as a sustainable base for the fabrication of green catalysts. In this context, we hypothesize that the CAF-capped nanomaterials may provide a significant input for the processes related to the catalytic hydrogenation of nitroaromatic compounds (NACs). NACs belong to major organic pollutants that reveal a toxic and carcinogenic character [10]. As such, their catalytic hydrogenations over the metallic NPs gain considerable attention [11].

Up to date, a variety of NPs, including AuNPs [12], PtNPs [13], PdNPs [14], AgNPs [15], and many more, were reported to catalytically reduce NACs under mild conditions. These NPs, however, are often synthesized in multi-stage processes, and with the use of compounds that often are toxic [14]. For this reason, we presume that CAF may offer a unique synthesis approach in which it may serve as a green and renewable reducing and capping agent for the metallic NPs, further aimed at catalyzing the processes related to the NACs’ hydrogenation.

The application of CAF for the synthesis and stabilization of metallic NPs is based on the premise that O and N chelating atoms contribute to the reduction of metal ions and to the further stabilization of the resultant NPs. In this premise, the π-electrons of CAF functional groups reduce metal ions or charged metal complexes, simultaneously being absorbed on the newly formed NPs and preventing in this way their agglomeration [16,17]. In the case of CAF, functional groups that can possibly take part in the formation/stabilization of NPs are C=O, –C=N, –CH_3_, and –N-C=O [17]. The described approach may be very tempting since there is no need for additional chemicals, about which a number of literature studies report, where the above-described mechanism of the NPs’ synthesis and their further stabilization was achieved by a variety of natural extracts containing compounds like phenols, polyphenols, alkaloids, terpenoids, and flavonoids [16,17,18,19]. Although the above-mentioned applications were successful, the use of CAF may yet offer another perspective for green NP synthesis; this compound may be derived from already named secondary sources, like coffee grounds, tea leaves, and cocoa beans [1,2,3]. Based on this, the CAF-based synthesis of metallic NPs may be recognized not only as a green and renewable approach but also a fully sustainable way to fabricate novel catalytical nanomaterials.

To the best of our knowledge, the approach to the synthesis of nanocatalysts for the hydrogenation of NACs only with CAF and no other reducing agents has never been reported in the literature. Further, because CAF is nontoxic and can be obtained from secondary sources, its use for the preparation of nanocatalysts may be considered as a step in achieving the sustainability of NAC hydrogenation. Therefore, within the present study, we propose for the first time nanocatalysts based on ReNPs, synthesized and stabilized by CAF derived from coffee and coffee grounds. It is presumed that, by combining ReNP synthesis with CAF derived from the natural product and its waste, it will be beneficial for the catalytical processes related to the hydrogenation of NACs on two different levels. The first one contributes to the economic aspects because Re is much cheaper than noble-metal-based solutions [20,21,22]. The second one contributes to ecological aspects because the use of CAF enables one to omit synthetic and/or toxic reducing agents, further allowing one to employ the CAF-containing wastes for the ReNP synthesis [12,23]. Within this idea, the green and sustainable synthesis provided by CAF will result in a new and sustainable catalyst whose effectiveness will be verified for the model reaction of the 4-nitrophenol (4-NP) to 4-aminophenol (4-AP) hydrogenation.

## 2. Results and Discussion

### 2.1. Catalytic Hydrogenation of 4-NP over CAF-Stabilized ReNPs

**Figure 2:**
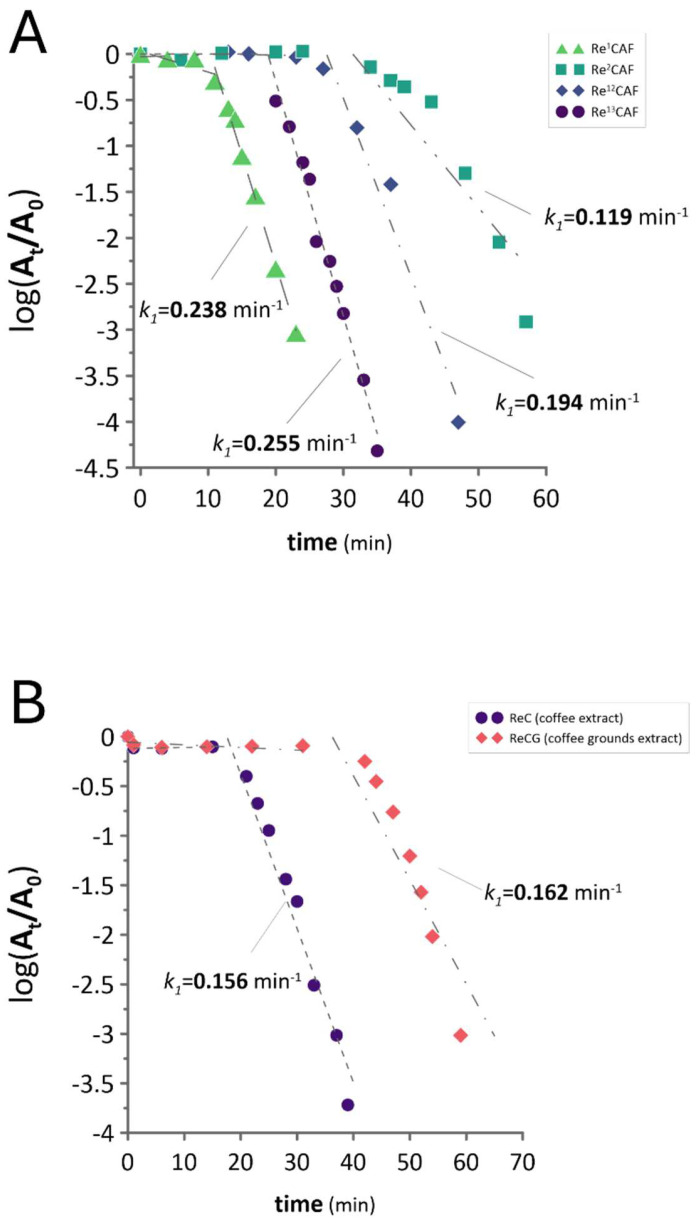
The catalytic activity of (**A**) ReNPs obtained using the synthetic CAF solutions and (**B**) ReNPs obtained using the solutions of CAF extracted from coffee and coffee grounds.

For the assessment of the kinetic behavior of the CAF-stabilized ReNP catalysts, tests on the catalytic activity were carried out in two steps. In the first step, ReNPs were synthesized using synthetic CAF solutions containing 1, 2, 12, and 13 mg CAF L^−1^, whose concentration matched the CAF concentration in the diluted water solutions of CAF obtained after the alkaline extraction of coffee and coffee grounds. This allowed us to optimize the CAF concentration required for the preparation of the most efficient ReNP catalysts. Figure 2A displays the pseudo-first-order kinetic plots for the catalytic hydrogenation of 4-NP over these catalysts.

These four catalysts, i.e., Re^1^CAF, Re^2^CAF, Re^12^CAF, and Re^13^CAF, served as a model to check the CAF’s ability to synthesize the ReNP-based catalysts. Based on the data presented in Figure 2A, there was no pattern linking the catalytic activity of the CAF-stabilized catalysts with the CAF concentration. The k_1_ values of 0.238, 0.119, 0.194, and 0.255 min^−1^, respectively, were established for the Re^1^CAF, Re^2^CAF, Re^12^CAF, and Re^13^CAF catalysts. Interestingly, the greatest catalytic activities were observed in the cases where the lowest and highest concentrated CAF solutions were used. It could be assumed that the higher the CAF concentration is, the more efficient the reduction of the Re(VII) ions becomes (a higher concentration means more electron-donor atoms). This could suggest why the ReNPs obtained with the aid of a 13 mg L^−1^ CAF solution (Re^13^CAF) were the most efficient. However, the opposite observation carried out for the ReNPs obtained with the aid of a 1 mg L^−1^ CAF solution might suggest that there was also another factor involved. The second-greatest k_1_ values in the case of the above-mentioned catalyst (Re^1^CAF) suggested that the low CAF concentration could make the resultant ReNPs more accessible in the catalytic reduction process. Both of these observations tended to the conclusion that the synthesis of an efficient catalyst must follow one of the two routes. The first route should include the efficient Re(VII) reduction and the second one should produce the accessible ReNPs.

Nevertheless, all of the catalysts led to an almost complete (93–99%) 4-NP hydrogenation. Each catalyst also displayed an induction period in the initial stages of the reaction. According to the recent work of Neal et al. [24], the catalytic reduction of 4-NP in the presence of NaBH_4_ may involve a side reaction in which the synthesized 4-AP can be re-oxidized back into 4-NP once desorbed from the surface of a catalyst [24]. So-produced 4-NP again reabsorbs onto the catalyst surface and is subjected to reduction once again. These two processes are opposed to each other, and, as such, in spectrophotometric terms, the reaction may appear to be stalled. The side reaction can occur until O_2_ is present in the reaction mixture and finally fades as the BH_4_^−^ ions scavenge O_2_, which results in the formation of H_3_BO_3_ as was proposed in recent studies [24,25,26]. Hence, while the so-occurring induction period comes to an end, the reduction of 4-NP advances. In the present study, however, different induction periods were observed. Shorter ones were shown by the samples obtained using 1 and 13 mg L^−1^ CAF solutions (for the Re^1^CAF and Re^13^CAF, respectively). With the solutions containing 2 and 12 mg L^−1^ of CAF, longer inductions were achieved, reaching up to 30 min of the reaction. This observation was also directly linked with the pseudo-first-order catalytic activity determined for the data points where the model became linear (see Figure 2A for details). The samples revealing longer induction periods (Re^2^CAF and Re^12^CAF) also revealed smaller k_1_ values. Because this could not be linked with the CAF concentration, it was likely to be related to the state of ReNPs. This conclusion will be further verified in the manuscript.

Based on the k_1_ values displayed in Figure 2A, two concentrations of CAF were defined as suitable for preparing the ReNP catalysts, i.e., 1 and 13 mg L^−1^. Therefore, the water solutions containing CAF extracted from coffee and coffee grounds were appropriately diluted so as to prepare the water solutions containing 13 and 1 mg L^−1^, respectively. Both of these solutions were then used in the same way as the synthetic CAF solutions to synthesize the ReNP catalysts, i.e., ReC and ReCG, consequently. Next, the catalysts ReC and ReCG were used in the catalytic hydrogenation of 4-NP, and the results of this are displayed in Figure 2B.

### 2.2. The Role of CAF in Synthesis and Capping of ReNPs

**Figure 3:**
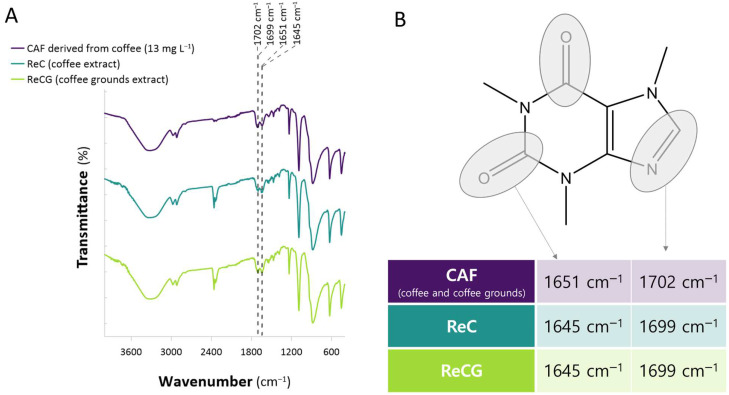
(**A**) FT-IR spectra of CAF solution derived from coffee (13 mg L^−1^ CAF) and the CAF-stabilized ReNPs (ReC, ReCG), (**B**) the identification of the characteristic bands shifts.

To verify the role of CAF in the synthesis and capping of ReNPs, an FTIR analysis was carried out. The spectra of the CAF extracts used for the synthesis of ReNPs (13 and 1 mg L^−1^ CAF in water) were measured before and after the addition of the Re(VII) solution, and these are displayed in Figure 3A. Because the spectra recorded for both CAF derived from coffee and coffee grounds were exactly the same, here, only the first one is referenced and compared to the samples ReC and ReCG.

Based on the recorded spectrum of CAF, two characteristic bands were found. These were associated with the carbonyl C=O and C–N groups present in the CAF structure (Figure 3B) [27]. The original position of these bands was 1651 and 1702 cm^−1^, respectively. After the addition of the Re(VII) ions and the capping of the resultant ReNPs, these bands shifted toward lower frequencies, i.e., 1645 and 1699 cm^−1^ (Figure 3), respectively. It was previously recognized that such slight band shifts could be attributed to the complexation of the metal ions and metallic nanoparticles by the functionalities in organic compounds. Such a phenomenon was already observed for a variety of metallic species, including Au, Pt, Pd, Re, and Sr [28,29,30,31,32,33]. Further, it has also been established that CAF reveals an ability to bind metal ions [34]. Based on this, it could be stated that the N- and O-bearing functionalities present in CAF might indeed play the role of a molecular rector for the synthesis and stabilization of ReNPs.

Besides the observed changes in the FT-IR spectra of CAF characteristic groups, other shifts could also be identified. These included the C-H stretching (from aromatic) vibrations at 2954 cm^−1^, the –CH_3_ bending vibrations at 1233 cm^−1^, the skeletal C-C vibrations at 1072 cm^−1^ and 861 cm^−1^, and the C-H bending vibrations at 643 cm^−1^. All of them shifted toward the lower wavenumbers as a result of the ReNP capping (Figure 3A). Besides these, the spectra displayed a couple of bands coming from the C-N stretching vibrations from tertiary amines (1356 and 1284 cm^−1^) [35,36]. They were shifted as well.

### 2.3. CAF-Stabilized ReNP Phase Identification

**Figure 4:**
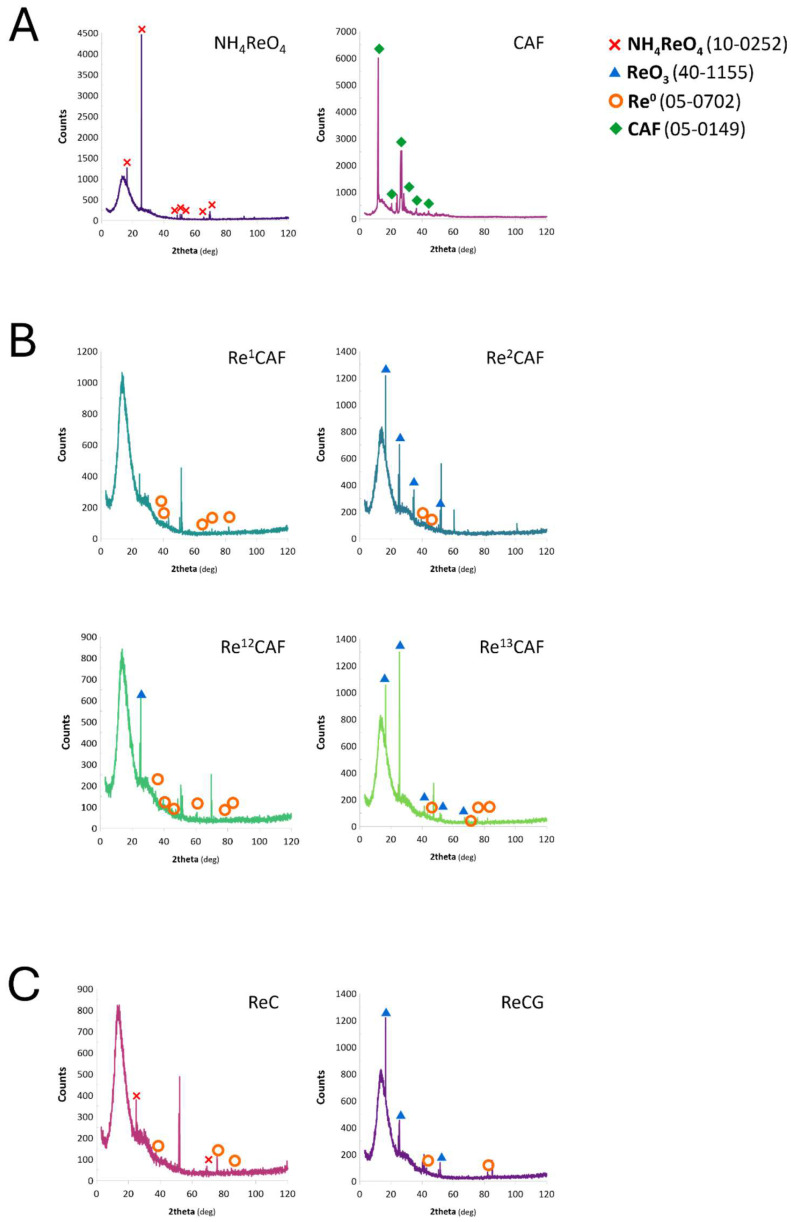
The XRD spectra of (**A**) NH_4_ReO_4_ and CAF; (**B**) ReNPs obtained using the synthetic 1 and 2 mg L^−1^ CAF solutions (Re^1^CAF and Re^2^CAF); (**C**) ReNPs obtained using the water CAF solutions after the extraction of coffee and coffee grounds (ReC and ReCG).

Because of the observed differences between pseudo-first-order rate constants of the catalysts obtained using different CAF solutions (synthetic and those achieved after the alkaline extraction), it was hypothesized that there must be differences in the characteristics of ReNPs. To verify this hypothesis and to reveal the phase composition of the obtained samples, an XRD analysis was performed and the diffractograms were analyzed (see Figure 4).

The XRD patterns were recorded for the samples of selected catalysts (Re^1^CAF, Re^2^CAF, ReC, RCG), as well as a NH_4_ReO_4_ powder crystallized from the solution used for ReNPs synthesis (1000 mg Re L^−1^) and evaporated CAF standard solution (13 mg L^−1^). The latter ones were used as the references, allowing us to determine the differences that occurred after mixing the precursor with CAF. Based on the patterns displayed in Figure 4, it can be stated that Re was found to be in three different forms. These included Re(VII) as NH_4_ReO_4_, Re(VI) as ReO_3_, and Re^0^, as was confirmed by comparing the observed reflexes with those reported in databases for pure metallic Re (05-0702), ReO_3_ (40-1155), and NH_4_ReO_4_ (10-0252) from the database ICDD PDF-2.

When comparing the phase composition of the catalysts obtained using the synthetic CAF solutions (Figure 4B), it could be concluded that the XRD patterns revealed the phases assigned to ReO_3_ and Re^0^. The best catalyst, Re^13^CAF (k_1_ = 0.255 min^−1^), contained a mixture of ReO_3_ and Re^0^, while the second-greatest k_1_ catalyst, Re^1^CAF (k_1_ = 0.235 min^−1^), contained only Re^0^ (Figure 4B). It must be noted, however, that the latter one exhibited the shortest induction period of the 4-NP reduction (Figure 2). In turn, blends of ReO_3_ and Re^0^, similar to those received for the best catalyst, were also observed in the case of the worst catalysts, Re^2^CAF and Re^12^CAF (k_1_ = 0.194 and 0.119 min^−1^, respectively). The difference was, however, that these two catalysts showed much smaller, even negligible, peaks assigned to Re^0^, and the count numbers assigned to ReO_3_ were smaller as well (Figure 4B). Based on these observations, and combining them with the observed catalytic activities (see Figure 2A), it was concluded that (1) the lower the Re oxidation state is, the shorter the induction time observed, and (2) the greater the count number of reduced forms of Re (being the result of the concentration), the greater the catalytic activity becomes. These observations are consistent with our previous studies [37,38], where greater catalytic activities in the reduction of NACs were revealed by ReNPs containing greater shares of reduced forms of Re. On the contrary, if nanoparticles contained more Re at greater oxidation states, both induction periods as well as catalytic activities negatively affected the catalytic hydrogenation of NACs. As a result, based on this, as well as the present studies, the determined catalytic activities of various forms of Re could be arranged in the following order: Re^0^ > Re^+4^ > Re^+6^ > Re^+7^ [37,38].

This could further suggest that, as stated above, the CAF concentration itself does not influence the catalytic activity of the ReNP catalysts. It actually influences the production of different Re oxidation states, and these, in turn, define the catalytic activity of the resulting ReNP catalysts. At this point, it must also be noted that a control experiment of catalytical reduction was carried out using NH_4_ReO_4_. Because the catalytical mixture involves the application of NaBH_4_ as the reduction bearer, the hydrogenation of 4-NP was completed. However, such a catalyst not only suffered a significant induction period but also revealed much smaller first-order rate constants (0.0019 min^−1^) compared to the CAF-stabilized Re.

These conclusions were also supported by the results of the 4-NP hydrogenation over the catalysts obtained using the water CAF solutions extracted from coffee and coffee grounds (Figure 2B). The sample ReC, mainly being the phases associated with Re^0^ (Figure 4C), also showed a significantly shorter induction time for the 4-NP reduction (Figure 2B) compared to the sample ReCG consisting of a blend of the Re^0^ and ReO_3_ phases (Figure 4B). This might suggest that the water solution of CAF extracted from coffee and coffee grounds could consist of a variety of other reducible moieties with electron donor atoms. This was further supported by the difference in the catalyst ReCG characteristics and behavior. CAF extracted from coffee grounds may not be able to perform the same reduction as CAF extracted from coffee.

### 2.4. CAF-Stabilized ReNP Morphology

**Figure 5:**
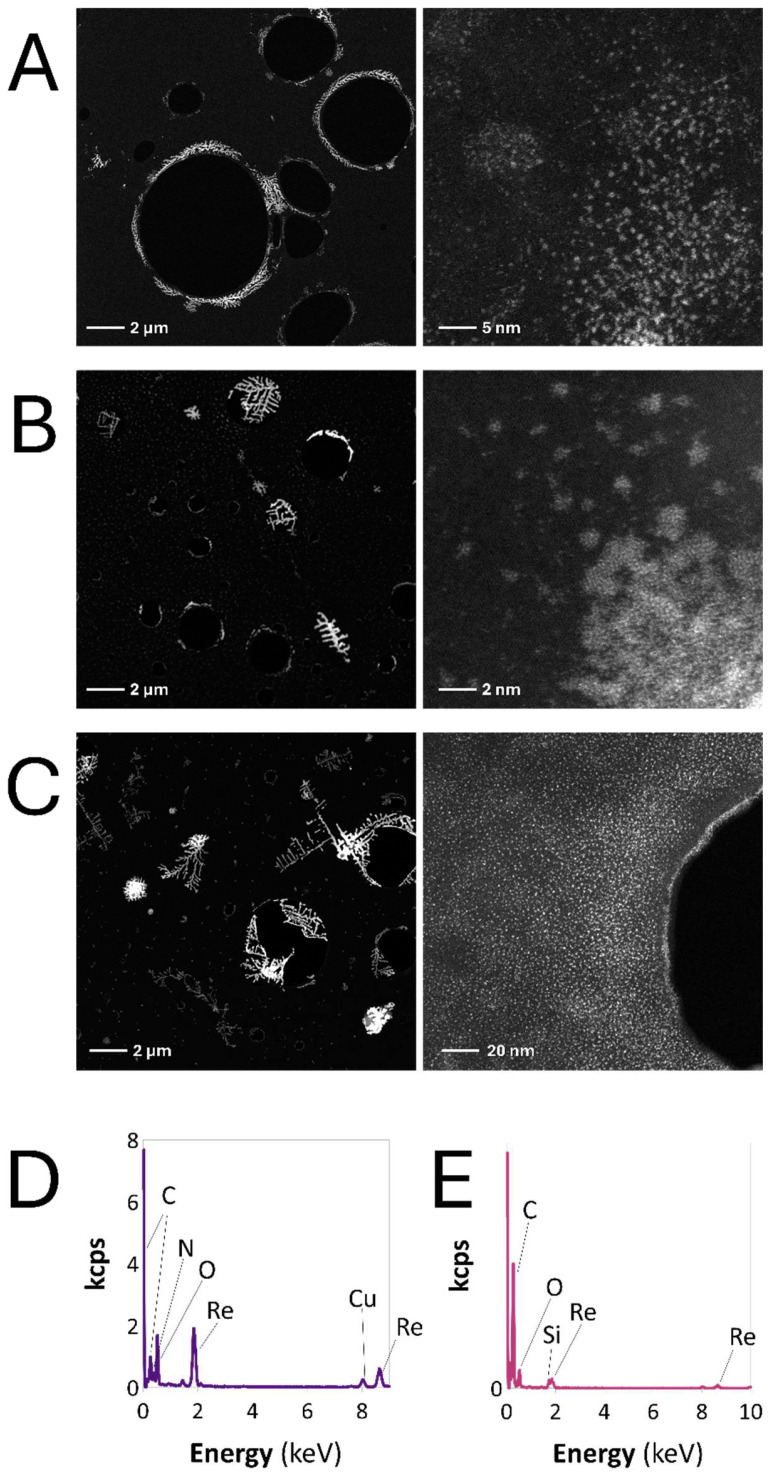
The HRTEM/HAADF images of (**A**) Re^13^CAF, (**B**) ReC, and (**C**) ReCG catalysts. The EDX spectra of (**D**) Re^13^CAF and (**E**) ReC.

The obtained CAF-stabilized ReNP catalysts revealed excellent catalytic activity in the reduction of 4-NP. Based on our experience with Re-based nanomaterials, we hypothesized that this ability of ReNPs arises from the unique properties of Re and its ability to fabricate particularly small structures [39]. For this reason, the morphology of the catalysts Re^13^CAF, ReC, and ReCG was investigated by an HRTEM/HAADF analysis. The resultant photographs and the EDX spectra are shown in Figure 5.

Based on the XRD pattern of CAF (Figure 4A), it was expected that the HRTEM analysis would display the crystalline characteristics of the CAF-containing samples. Indeed, as can be seen in Figure 5, crystalline, dendrimer-like structures were observed, which may be associated with CAF itself. Furthermore, the EDX spectra displayed in Figure 5D,E display the presence of C, O, and N atoms, which can be associated with CAF functionalities (Figure 1). This, combined with the observed shifts in the FT-IR spectra (Figure 3A), may suggest that CAF indeed took part in the reduction of Re(VII).

The ReNP characteristics of the samples obtained in the present work are consistent with our previous studies [37,39]. All samples contained very small Re structures, consisting of groups of Re atoms. Even the structures that could be named as a “nanoparticle” still evidently consisted of separate, much smaller Re structures (Figure 5A–C). Simultaneously, these Re “apparent” NPs were well dispersed. Based on data given in Figure 5, the morphology of Re reduced by CAF was very similar in each sample; hence, the above-stated conclusion about the relationship between the Re phase composition and the catalyst activity seems to be valid.

### 2.5. CAF-Stabilized ReNPs Versus Other Catalysts for NAC Hydrogenations

The catalytic activity of synthesized ReNPs was compared with other noble metal NPs synthesized in a “green way” similarly as in the present study, i.e., using natural-originated products and no additional reducing agents. Within this concept, natural-derived compounds or plant extracts were already reported to serve as the reducing agents and stabilizers. These are summarized in Table 1. Based on the collected data, ReNPs obtained in this study showed an outstandingly high k_1_ value (0.255 min^−1^) of the 4-NP reduction compared to others. Compared to others, the obtained k_1_ value was up to 2500 times higher than the respective values achieved for AuNPs [40], AgNPs [41], and PtNPs [42]. The latter one was also obtained using a CAF extract derived from *Coffea arabica*, but resulted in a catalyst characterized by a k_1_ value of 0.001 min^−1^ [42]. The best result found in the literature on “green” NPs applied for 4-NP hydrogenation was PdNPs, obtained with the aid of an extract of *Sterculia acuminata* seeds. However, the k_1_ value exhibited thereof was 0.179 min^−1^, which is still not as good as the value achieved with the ReNPs obtained in the present study (Table 1).

## 3. Materials and Methods

### 3.1. Materials, Methods of Analyses, and Instrumentation

Caffeine (CAF, ≥99%) used for preparing standard solutions as well as CHCl_3_ (analytical grade) used for the CAF extraction were purchased from Sigma-Aldrich (Poznań, Poland). Sodium hydroxide (NaOH, ≥99%) was purchased from Merck (Poznań, Poland). Coffee beans, distributed by one of the globally known coffee companies, were bought in a store (Wroclaw, Poland) and then used to make a couple of infusions in a Saeco GranAroma (Philips, Amsterdam, Netherlands) coffee machine, model SM6585/00 (operation conditions: 15 bars and 90–98 °C water temp.). Wasted coffee grounds were then collected, dried, and stored in a fridge. Subsequently, some coffee beans were ground to obtain coffee powder, which was also stored cold. Redistilled water was used throughout all experiments, excluding coffee brewing, where tap water was used. Ammonium perrhenate (NH_4_ReO_4_, ≥99%) for the catalyst synthesis as well as sodium tetra borohydride (NaBH_4_, ≥98%) and 4-NP (≥99%) for the catalytical activity study were acquired from Sigma-Aldrich (branch Poland) and used as received.

To measure the absorbance of caffeine in chloroform solutions, a Thermo Scientific UV-Vis spectrophotometer (GENESYS 10S, Argenta, Poznań, Poland) was used. The absorbance measurements of 4-NP, carried out during the tests on the catalytic activity, were performed using an Analityk Jena UV-Vis spectrophotometer (SPECORD PLUS210, MS Spektrum, Warszawa, Poland). Fourier transformation infrared spectroscopy (FT-IR, Jasco FT-IR-4700, Medson, Paczkowo, Poland) and X-ray powder diffraction (XRD, Philips X’PERT, Amsterdam, Netherlands), as well as high-resolution transmission electron microscopy (HRTEM, FEI TITAN^3^, Thermo Scientific, Waltham, MA, USA), were used for determining catalyst characteristics. For the XRD analysis, the samples were prepared by drop-casting of suspension solution followed by solution evaporation. In the case of HRTEM analysis, the instrument was equipped with a selected area electron diffractometer (EDX) and a high-angle annular dark-field (HAADF) detector operating in the scanning transmission electron microscopy (STEM) mode.

### 3.2. Caffeine Extraction

The alkaline extraction of CAF from coffee (1.0 g) and coffee grounds (1.0 g) was made with CHCl_3_ and was repeated 4 times, while the extracts originating from all steps were combined. The extraction process started by placing the samples into separatory funnels. Next, 10 mL of a 0.2 mol L^−1^ NaOH solution was used to moisten the samples and alkalize them. For the first step of extraction, 20 mL of CHCl_3_ was added and the funnels were agitated vigorously for 7 min. The organic phase was collected into volumetric flasks (50.00 mL). Then, the extraction procedure was repeated 3 times more; for each step, 5.00 mL of CHCl_3_ was used. After all steps, the volumetric flasks with the extracts were made up to the mark with CHCl_3_. To perform the spectrophotometric measurements, the prepared sample extracts were 25-fold diluted. Finally, the absorbance measurements of the diluted sample extracts were taken within the wavelength range of 240–340 nm to assess the CAF concentration.

Before the synthesis of the catalysts, the sample extracts were evaporated, and the CAF residues left were dissolved in water to obtain their aqueous solutions. The obtained water CAF solutions were additionally filtrated through syringe filters (pore size 0.45 µm). As a result, two water CAF solutions were obtained: one derived from coffee and the other one from coffee grounds. The CAF concentration spectrophotometrically determined in these solutions was 161.4 ± 6.7 mg L^−1^ (coffee) and 23.8 ± 1.7 mg L^−1^ (coffee grounds).

### 3.3. Synthesis of CAF-Stabilized Re Catalysts

For the catalyst synthesis, a stock water solution of Re(VII) (1000 mg L^−1^) was prepared. Then, the synthesis and capping of the resulting ReNPs were performed by mixing 9.0 mL of the Re(VII) stock solution and 1.0 mL of the prepared water CAF solutions. Afterwards, the mixtures were left for 48 h on an orbital shaker. This equilibration was defined as enough for the reduction of the Re(VII) ions [39] with simultaneous formation and capping of ReNPs.

For the purpose of this study, two series of catalysts were prepared. The first series was synthesized with the use of the CAF standard solutions, in which the CAF concentration was 1, 2, 12, and 13 mg L^−1^. The selection of CAF concentrations was motivated by the fact that the absorbance of caffeine (CAF) in the UV-Vis region (240–340 nm) overlapped with the absorbances of 4-nitrophenol (318 nm) and the products of its reduction, namely, 4-aminophenol (295 nm). For this reason, CAF concentration of 13 mg L^−1^ was defined as the maximum value that would be suitable for carrying out the catalytical reaction. This strategy aligned with the other motivation of using the least possible CAF, which, in this context, is considered as a resource. Hence, applying 1 and 13 mg L^−1^ concentrations of CAF met both of these criteria. The other concentrations, 2 and 12 mg L^−1^, were presented to highlight the link between CAF and rate constants of catalytical reactions. These catalysts were coded as Re^1^CAF, Re^2^CAF, Re^12^CAF, and Re^13^CAF, and were used to optimize the CAF concentration required for the synthesis with respect to the catalytic activity of the resultant catalyst. To do so, the so-obtained catalysts were used for the hydrogenation reaction of 4-NP (see Section 2.1 for details). Based on the calculated first-order rate constants (k_1_, min^−1^) and the yields (%) of the 4-NP reduction, the optimal CAF concentration was defined. As such, the second series of catalysts was synthesized with the use of appropriately diluted water solutions of CAF extracted from coffee and coffee grounds. Because the extracts were characterized by different concentrations of CAF (see Section 2.1 for details), the extract from coffee was used to obtain 13 mg L^−1^ CAF solution, while the extract from coffee grounds was used to prepare 1 mg L^−1^ CAF solution.

### 3.4. Catalytic Hydrogenation of 4-NP

The catalytic activity was measured by carrying out a reduction of 4-NP to 4-AP over the Re-based nanocatalysts. The reaction performance was monitored by recording the spectra in the wavelength range of 250–600 nm. First, the absorbance was measured only for a 4-NP solution (2.5 mL), and then 0.2 mL of a 0.1 mol L^−1^ NaBH_4_ solution was added and the spectrum was recorded. Finally, 0.3 mL of a catalyst solution was added, which initiated the reaction—the spectra were recorded next at fixed periods of time. The catalytic reaction was monitored by measuring the absorbance at λ_max_ 400 nm, which was assigned to the –NO_2_ group in the 4-nitrophenolate anion. These absorbance values were used for determining (1) the pseudo-first-order rate constants (k_1_) and (2) the yields (%) of the 4-NP reduction. The first one was calculated based on the slope of the ln*A_t_*/*A*_0_ vs. *t* plots, where *A_t_* is the absorbance at time *t* and *A*_0_ is the absorbance at the beginning of the process. The second one was calculated based on the mass balance. All calculations were performed assuming that the *A_t_/A*_0_ ratio is proportional to the 4-NP concentration.

## 4. Conclusions

In this work, we successfully synthesized green Re-based catalysts using waste-derived CAF that act as a reductor and stabilizer at once. The synthesized CAF-stabilized ReNPs revealed the enhanced catalytic activity, specifically for the hydrogenation of 4-NP to 4-AP. Within this concept, this research repurposes food industry waste and also contributes to green chemistry and sustainable catalyst development.

It was confirmed that CAF extracted from coffee and coffee grounds can effectively reduce Re(VII) ions and stabilize the resulting ReNPs. This dual role of CAF, owing to its rich electron-donating N and O atoms, underscores its potential as a sustainable alternative to the conventional, often toxic, reducing agents. The so-obtained ReNPs show a significant catalytic efficiency in the hydrogenation of 4-NP. Among all tested samples, those prepared with the solutions of CAF at 1 and 13 mg L^−1^ are characterized by the highest pseudo-first-order rate constants. This variability in the catalytic activity suggests that both high and low CAF concentrations can effectively produce active catalysts, likely due to differences in the accessibility and reduction efficiency of ReNPs.

The FT-IR spectra indicate some shifts in the characteristic bands of CAF upon the ReNP formation, confirming the interaction between CAF functional groups and the Re ions. In turn, the XRD analysis revealed that the phase composition of ReNPs, particularly the presence of Re^0^ and ReO_3_, is crucial for the catalytic performance. The morphology analysis further confirmed that CAF-derived ReNPs are well dispersed and consist of very small Re structures, contributing to their high catalytic activity.

The use of waste-derived CAF not only addresses the important environmental concerns related to food waste but also presents an economically viable route for producing Re-based catalysts. The cost-effectiveness of Re over more expensive noble metals adds to the practical applicability of this synthetic approach. The so-obtained ReNPs show greater catalytic activity compared to other metallic NPs. In this context, undertaken future research can focus on the optimization of the CAF extraction methods and the potential of other waste-derived compounds in ReNP synthesis, ultimately contributing to a more sustainable and eco-friendly chemical industry.

## Figures and Tables

**Table 1:** The comparison of the catalytic activity of different metallic NPs.

	4-NP Hydrogenation k_1_ (min^−1^)	NP Synthesis Methodology	Ref.
AuNPs	0.0001	Aspartame (reducing agent) mixed with a HAuCl_4_ solution	[40]
AgNPs	0.0002	Breynia rhamnoides steam extract (as reducing agent) mixed with a AgNO_3_ solution	[41]
PtNPs	0.001	Coffea Arabica seed extract (as reducer) mixed with a H_2_PtCl_6_ solution	[42]
PdNPs	0.179	Sterculia acuminata seed extract (as reducer/stabilizer) mixed with a PdCl_2_ solution	[43]
ReNPs	0.255	CAF solutions extracted from coffee and coffee grounds mixed with a NH_4_ReO_4_ solution	Present study

## Data Availability

All data associated with this work are available under a permanent identifier: https://doi.org/10.18150/8ESUOT.
